# Data describing the effects of potassium channels modulators on outward currents measured in human lymphoma cell lines

**DOI:** 10.1016/j.dib.2020.106668

**Published:** 2020-12-21

**Authors:** Alberto Montalbano, Cesare Sala, Chiara Abrardo, Nicoletta Murciano, Farhad Jahanfar, Massimo D'Amico, Francesco Bertoni, Andrea Becchetti, Annarosa Arcangeli

**Affiliations:** a Department of Experimental and Clinical Medicine, University of Florence, I‑50134 Florence, Italy; b DIVAL Toscana Srl, Via Madonna del Piano 6, Sesto Fiorentino, I-50119 Firenze, Italy; c Department of Medical Biotechnologies, University of Siena, I-53100 Siena, Italy; d Institute of Oncology Research, Faculty of Biomedical Sciences, USI, via Vela 6, CH-6500 Bellinzona, Switzerland; e Oncology Institute of Southern Switzerland (IOSI), Ospedale San Giovanni, CH-6500 Bellinzona, Switzerland; f Department of Biotechnology and Biosciences, University of Milano-Bicocca, Piazza della Scienza 2, I-20126 Milano, Italy

**Keywords:** Human lymphomas, Potassium channels, Kv1.3, KCa3.1, Patch-clamp

## Abstract

In the present work, applying the whole-cell patch-clamp technique in voltage clamp mode, we have investigated the effects of different drugs, such as riluzole, Psora-4 and Tram-34, on the potassium currents in four human lymphoma cell lines. We focused on outward currents mediated by two potassium channels (Kv1.3 and KCa3.1), which are known to play a key physiological role in lymphoid cells. The currents were evoked by voltage ramps ranging from -120 mV to +40 mV and the conductance of the two potassium channels was measured between +20 mV and +40 mV, both in the absence and in the presence of the specific blockers Psora-4 (Kv1.3; 1 µM) and Tram-34 (KCa3.1; 1 µM). The effect of the latter was tested after KCa3.1 channels were activated by riluzole 10 µM. Taken together, these data could be useful as an indication of the functional characteristics of the potassium channels in human lymphomas and represent a starting point for the study of potassium conductance in cellular models of these tumors.

## Specifications Table

**Table:** 

Subject	Cancer Research
Specific subject area	Potassium channels electrophysiology
Type of data	Raw and graph
How data were acquired	Data were acquired through a Multiclamp 700A equipment, digitized with a Digidata 1322A (Molecular Devices, Sunnyvale, California, USA) and the recordings were low-pass filtered at 3 KHz and digitally sampled at 25 KHz.
Data format	Raw
Parameters for data collection	Sample: model cells of human lymphomas Parameters: voltage-clamp whole-cell recordings of potassium currents evoked by −120 mV to +40 mV voltage ramps. Potassium channel conductance was measured between +20 mV and +40 mV.
Description of data collection	Epstein-Barr virus-infected B lymphocytes, diffuse large B cell lymphoma cell lines (WSU-DLCL2, SU-DHL-4, SU-DHL-6) were cultured in RPMI 1640 and then on the experiment day plated on 35 mm Petri dishes for 1 hour to obtain the cell adhesion before performing whole-cell voltage-clamp electrophysiological recordings.
Data source location	Department of Experimental and Clinical Medicine, University of Florence, I‑50,134 Florence, Italy
Data accessibility	Repository name: Zenodo Direct URL to data: Kv1.3: DHL-4: https://zenodo.org/record/4289852#.X843PulKho4 DHL-6: https://zenodo.org/record/4289853#.X843FOlKho4 WSU-DLCL2: https://zenodo.org/record/4289856#.X84xrelKho4 EBV: https://zenodo.org/record/4289858#.X8428ulKho4 KCa3.1: DHL-4: https://zenodo.org/record/4289859#.X84 × 4-lKho4 DHL-6: https://zenodo.org/record/4289869#.X842z-lKho4 WSU-DLCL2: https://zenodo.org/record/4289872#.X84yL-lKho4 EBV: https://zenodo.org/record/4289870#.X84yfulKho4

## Value of the Data

• A functional role of ion channels in different tumor types is increasingly recognized. The role of potassium channels has been widely studied in B lymphocyte physiology but not in lymphomas. Our dataset represents a start point for the study of potassium channels in cellular models of human lymphomas.
• The shared data can be used by researchers as a comparative model for studying the conduction properties of potassium channels in tumor cell lines and especially for non-solid tumors (i.e. lymphocyte-derived cancer cells).
• By providing a benchmark dataset the shared data are likely to be valuable in further development and refinement of ion channel conductance measurements on non-adhesive cell types.

## 1 Data Description

**Fig. 1:**
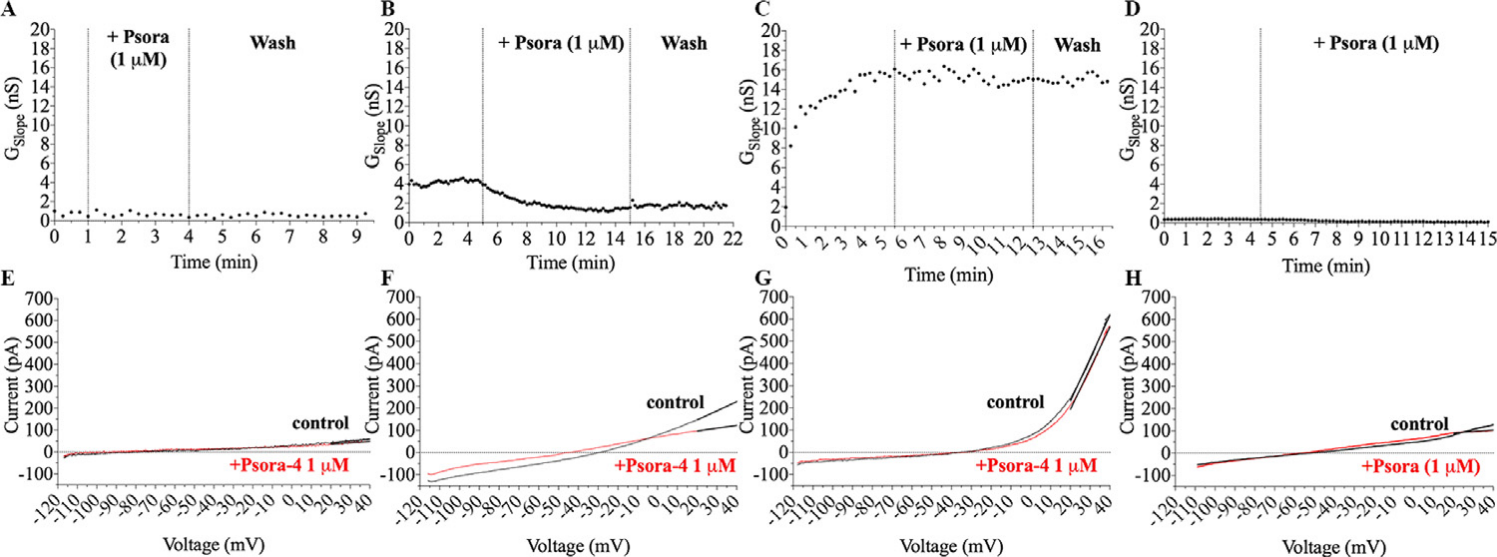
Representative time courses of Kv1.3 slope conductances measured in control conditions and in the presence of Psora-4 1 µM for (A) Epstein-Barr virus-infected B lymphocytes, (B) SU-DHL-4, (C) SU-DHL-6 and (D) WSU-DLCL2 respectively. Lower row: representative traces of the corresponding experiments in control (black) and in Psora-4 (red) for (E) Epstein-Barr virus-infected B lymphocytes, (F) SU-DHL-4, (G) SU-DHL-6 and (H) WSU-DLCL2 respectively.

In SU-DHL4 cell line the application of 1 µM Psora-4 produced a significant reduction of the slope conductance from 11.6 ± 9.8 nS to 9.2 ± 8.6 nS and we measured an average net Psora-sensitive conductance of 2.4 ± 2.5 nS (*p* = 0.01, *n* = 10). On the other hand on SU-DHL-6 cells the application of 1 µM Psora 4 produced a non significant reduction on the slope conductance (control: 18.1 ± 6.3 nS, treated: 17.2 ± 5.4 nS, *n* = 7; *t*-test). Similar results were obtained for WSU-DLCL2 (control: 1.3 ± 0.6 nS, treated: 0.7 ± 0.5 nS, *n* = 5) as well as EBV infected lymphocytes (control: 7.0 ± 6.3 nS, treated: 6.5 ± 6.7 nS, *n* = 8). Fig. 1 shows the time courses under control conditions and in the presence of Psora-4 (1 µM) representative of each cell line examined together with their relative current ramps.

**Fig. 2:**
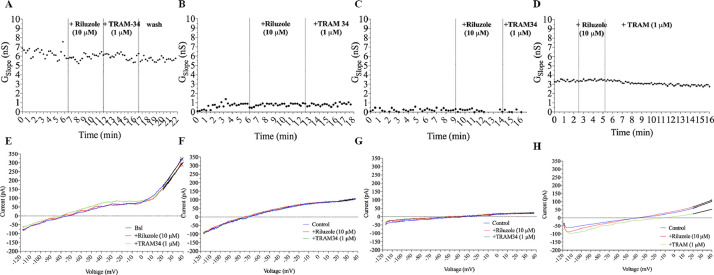
Representative time courses of KCa3.1 slope conductance measured on (A) Epstein-Barr virus-infected B lymphocytes, (B) SU-DHL-4, (C) SU-DHL-6 and (D) WSU-DLCL2 cell lines. No effects were observed in any cell line upon KCa3.1 riluzole-mediated activation (10 µM) nor in the presence of the specific blocker TRAM-34 (1 µM). Lower row: representative traces of the corresponding experiments in control (blue), riluzole 10 µM (red) and in TRAM-34 (red) for (E) Epstein-Barr virus-infected B lymphocytes, (F) SU-DHL-4, (G) SU-DHL-6 and (H) WSU-DLCL2 respectively. (For interpretation of the references to colour in this figure legend, the reader is referred to the web version of this article.)

- Application of the specific blocker Tram-34 (1 μM), following KCa3.1 activation by riluzole (10 μM), did not produced any significant effect in the studies cell lines. In SU-DHL4 cells, application of Tram-34 (1 µM) had no effects on the slope conductance (control: 1.6 ± 2.1 nS, treated: 2.0 ± 1.8 nS, *n* = 5; *t*-test) as for SU-DHL6 cells (control: 0.5 ± 0.3 nS, treated: 0.5 ± 0.4 nS, *n* = 7). Similar results were obtained also for WSU-DLCL2 (control: 2.1 ± 1.1 nS, treated: 1.0 ± 0.7 nS, *n* = 5) and EBV infected lymphocytes (control: 11.1 ± 4.0 nS, treated: 10.4 ± 4.7 nS, *n* = 5; *t*-test). Fig. 2 shows the time courses under control conditions, in the presence of the specific activator for KCa3.1 riluzole (10 µM) and subsequently of the specific blocker Tram-34 (1 µM) representative of each cell line examined.

- The raw data of the electrophysiological recordings are organized as follows in the Zenodo repository:

- Each folder is named with the name of the cell line and contains the .csv files related to the electrophysiological recordings. Moreover, it contains an .xls file (eg. DHL-4_Kv1.3_raw.xls). This .xls file contains the measured conductance values in the various experimental conditions (ctrl, drugs etc.) relative to each single cell of that cell line.
- Each .csv file corresponds to a cell of its relative cell line with the exception of files whose name ends with −1 or −2 (for example 191120_C1–1, 191120_C1–2). In these cases, the recording related to a cell has been divided in two files and −1 and −2 correspond to the first and second part of the recording, respectively.
- For each .csv file: column 1 is an index column representing the sample number (not useful for analysis purposes), column 2 (Time) reports in ms each of the sample-by-sample timepoints, column 3 (Vcmd) is the potential value in mV imposed during the protocol, from column 4 (Sw…) onwards the single sweeps of that recording are reported (column by column) and each cell of a sweep column corresponds to the current (pA) measured sample by sample.
- For the purposes of the analysis, the potential column (Vcmd) should be plotted on the x axis and one (or more) sweep column(s) on the y axis. So far it will be possible to display one (or more) ramps: from the Vcmd vs. Current plot it is then possible to perform a linear fit in the range between +20 and +40 mV. The slope of this fit corresponds to the conductance value for each ramp.
- The time interval between one sweep and the next one is 10 s (0.1666667 min), therefore it is possible to plot each single conductance value (measured as previously described) as a function of time and thus obtain the conductances time-courses as shown in the representative figures of this manuscript.

- Drugs administration time points for each experiment are as follows:

**Table:** 

Kv1.3 - DHL-4		
Cell	Control (min)	+Psora-4 1 µM (min)
191,120_C1	0–5	5–15
191,120_C2	0–3.5	3.5–13.5
191,125_C1	no delivery	no delivery
200,110_C11	0–3.5	3.5–11
200,110_C16	0–3	3–8
200,113_C1	0–5	5–10
200,113_C2	0–5.5	5.5–11
200,113_C3	0–4	4–9
200,120_C1	0–3.5	3.5–9.5
200,120_C3	0–2.5	2.5–7.5

Kv1.3 - DHL-6		

191,209_C1	0–4	4–14
200,117_C1	0–3.5	3.5–10
200,117_C2	0–5.5	5.5–12.5
200,117_C3	0–5.5	5.5–11.5
200,120_C5	0–3.5	3.5–10
200,120_C7	0–5.5	5.5–11.5
200,120_C8	0–5.5	5.5–10.5

**Table:** 

Kv1.3 - WSU-DLCL2		

191,120_C1	0–4.5	4–18
191,120_C2	0–4.5	4.5–14.5
191,121_C1	0–2.5	2.5–5.5
191,121_C4	0–2.5	2.5–10.5
191,128_C2	0–7.5	7.5–16.5

Kv1.3 - EBV		

200,108_C4	0–2	2–10
200,108_C5	0–3.5	3.5–13.5
200,109_C4	0–1	1–4
200,109_C7	0–1	1–4
200,109_C9	0–6	6–11
N200109_C2	0–5	5–10
N200109_C3	0–4.5	4.5–10.5
N200109_C4	0–3.5	3.5–8.5

**Table:** 

KCa3.1 - DHL-4			

Cell	Control (min)	+Riluzole 10 µM (min)	+TRAM-34 1 µM (min)

191,128_C5	0–5	5–10	10-end
191,209_C3	0–4	4–9	9-end
191,211_C6	0–12	12–17	17-end
191,212_C3	0–2.5	2.5–10.5	10.5-end
191,212_C4	0–6	6–12.5	12.5-end

KCa3.1 - DHL-6			

191,212_C1	0–3	3–6	6-end
191,212_C2	0–9	9–14	14-end
191,212_C5	0–5	5–12	12-end
191,216_C2	0–4	4–7	7-end
191,216_C4	0–3	3–6.5	6.5-end

KCa3.1 - WSU-DLCL2			

191,120_C1	0–4.5	4.5–10	10-end
191,120_C6	0–10	10–20	20-end
191,120_C8	0-end	no delivery	no delivery
191,121_C10	0–3	3–5	5-end
191,121_C8	0-end	no delivery	no delivery
200,117_C11	0–3	3–7	7-end

## 2 Experimental Design, Materials and Methods

Human lymphoma cell lines. Epstein-Barr virus-infected B lymphocytes were previously generated in our laboratory [1]. Epstein-Barr virus-infected B lymphocytes, the DLBCL (GC subtype) cell lines SU-DHL4 and SU-DHL6 were cultured in RPMI 1640 medium supplemented with 2 mM l-glutamine, 10% bovine calf serum (HyClone), 100 U/mL penicillin, and 100 µg/mL streptomycin. The DLBCL, SU-DHL4 and SU-DHL6 cell lines are derived from germinal center B-cell type diffuse large B-cell lymphoma, which is the most common type of B-cell lymphoma. These cell represent in vitro models to functionally study the ion channels expression pattern recently reported in clinical specimens [2].

Electrophysiological recordings. Cells were plated on the experimental day directly on plastic. Electrophysiological recordings has been performed at room temperature (∼25 °C) in the whole-cell configuration of the patch-clamp technique, after 1 hour in an incubator at 37 °C [3], [4], [5]. The patch pipettes were pulled from borosilicate glass capillary tubes, their resistance was 4–5 MΩ and their capacitances were manually compensated up to 90–95% after the reaching of a stable gigaseal. Experimental protocols and data acquisition has been performed with the Multiclamp 700A amplifier and pCLAMP 9.2 software (Molecular Devices, Sunnyvale, California, USA) has been used for data analysis. All the outward potassium currents were measured with a 25KHz sampling rate and a 3 KHz low-pass filter. Cells' identification and patch has been performed at 40x magnification with a Nikon Eclipse TE300 microscope (Nikon Instruments Inc.), equipped with a Photometrics CoolSNAP CF camera (Teledyne Photometrics, Tucson AZ). Cells membrane potentials were held at −80 mV and outward potassium currents were elicited by 200 ms voltage ramps (−120 to +40 mV) with an intersweep interval of 10 s [6]. The internal pipette solution for Kv1.3 conductances measurement contained (in mM): 130 *K*+ aspartate, 10 NaCl, 4 CaCl2, 2 MgCl2, 10 Hepes–NaOH, 10 EGTA, pH 7.3. The external solution, instead, contained (in mM): 130 NaCl, 5 KCl, 2 CaCl2, 2 MgCl2, 10 HEPES, 5 Glucose (EK = −80 mV), pH of 7.4. For KCa3.1 currents a different internal pipette solution was used (in mM): 145 *K*+ aspartate, 8.55 CaCl2, 2 MgCl2, 10 HEPES, 10 EGTA, pH 7.2. In this case, the external solution was a Na+ aspartate Ringer, containing (in mM): 160 Na+ aspartate, 4.5 KCl, 2 CaCl2, 1 MgCl2, 5 Hepes–NaOH, pH 7.4. The GSlope values have been measured offline between +20 and +40 mV potentials range with the Clampfit software. The difference between the elicited currents recorded in control condition and the ones recorded in the presence of Psora-4 (1 µM) represented the Psora-4-sensitive current (Kv1.3 current). The effects of the KCa3.1 specific inhibitor TRAM-34 at 1 µM, instead, has been assessed on the maximal KCa3.1 activation, induced by Riluzole (10 µM). The 1 µM concentration for both Psora-4 and TRAM-34 was chosen because of its capability of evoking a maximal effect and drugs has been applied for at leat 5 min. Resting membrane potential (VREST) values were measured in I-0 mode.

Statistics. Parametric tests were used for statistical analysis, i.e., paired and unpaired *t*-test. Data are reported as mean ± SD and median ± interquartile range (IQR). The normality of data distribution was checked with Kolmogorov–Smirnov test. Statistical analysis was performed using Prism 6 software (GraphPad Software, San Diego, CA, USA). All statistical tests were two tailed with a significance level of 0.05.

## Declaration of Competing Interest

The authors declare that they have no known competing financial interests or personal relationships which have, or could be perceived to have, influenced the work reported in this article.
